# Development of an automated, multiwell plate based screening system for suspension cell culture

**DOI:** 10.1186/1753-6561-5-S8-O9

**Published:** 2011-11-22

**Authors:** Sven Markert, Klaus Joeris

**Affiliations:** 1 Roche Diagnostics GmbH, Pharma Biotech Production and Development, Penzberg, Germany

## Introduction

The automation of cell culture processes becomes more important in the pharmaceutical industry due to compressed timelines and the need to develop more products more efficiently. This drive to develop new processes faster and more efficient requires a streamlined workflow.

Resource intensive approaches like the use of shake flasks limit the accessible design space for the development of highly productive processes or the characterization of established processes. Process automation provides the appropriate tools to address the following key points:

Increasing experimental throughput   ⇨   “Design of Experiments” using full factorial designs

Increasing process information     ⇨   improve process understanding (“Quality by Design”)

Automate repetitive manual work    ⇨   gain efficiency, focus on high value tasks

Improve reproducibility         ⇨   ensure robust processes

**Figure 1:**
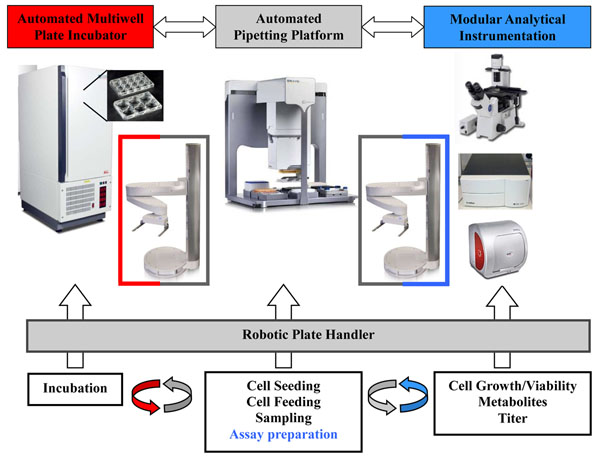
Schematic illustration of the developed robotic screening system prototype. Only the core system is shown with a robotic plate handler as key device connecting shaken cultivation, processing and analytical components.

A robotic plate handler based system was selected to meet the demands of a flexible, fast and modular screening system as presented in figure 1.

## Results

### Scale-up prediction

The comparability of results obtained with this new multiwell plate based culture system for suspension adapted cell lines plates and bioreactors had to be verified. It could be shown that 6-well plates were predictive for a scale-up to a 1000 L stirred tank reactor. The parameter profiles of viable cell density, lactate and antibody concentration were comparable in multiwell plates and bioreactors (2 L, 10 L and 1000 L). The plates can be used for process scale-up prediction.

### Media screening

An automated media blend screening was carried out in a second experiment highlighting another main area of application. Two seed trains of a CHO cell line, media blends of two growth media and two feeding strategies were screened in 120 wells on 6 well plates. A success rate of 100 % enabled the evaluation of all wells in terms of cell growth and productivity. An increase in viable cell density and product titer of about 20% in comparison to the reference process was achieved.

2 L bioreactor runs were performed to confirm these optimized parameters. A total of 6 bioreactor runs using the identified best combination of media blends, seed train and feeding strategy verified the predicted results from the multiwell plates and showed an increase in productivity of about 15 %.

## Conclusion and outlook

The developed robotic screening system is capable of performing a fully automated workflow consisting of incubation, sampling, feeding and near real-time analytics. The scalability from the mL-scale up to 1000 L scale could be shown. Furthermore, a successful screening application was carried out and an increase in product concentration could be achieved. This potential for a process improvement was confirmed in a bioreactor study. The robotic screening system has therefore been proven to be a suitable screening tool for process optimization.

Ongoing work is focusing on extending the analytical portfolio by including additional analytical methods and instrumentation. A second focus area is in the field of process characterization and process robustness studies.

